# Circulating Pro-Uroguanylin Levels In Children And Their Relation To Obesity, Sex And Puberty

**DOI:** 10.1038/s41598-018-32767-7

**Published:** 2018-09-28

**Authors:** Cintia Folgueira, Silvia Barja-Fernández, Patricia Gonzalez-Saenz, Cecilia Castelao, Rocío Vázquez-Cobela, Veronica Pena-Leon, Manuel Ruiz-Piñon, Felipe F. Casanueva, Carlos Dieguez, Rosaura Leis, Rubén Nogueiras, Luisa M. Seoane

**Affiliations:** 1 Fisiopatología Endocrina, Instituto de Investigación Sanitaria de Santiago de Compostela (IDIS), Complejo Hospitalario Universitario de Santiago de Compostela (CHUS/SERGAS), Santiago de Compostela, Spain; 2 Department of Physiology, CIMUS, Universidad de Santiago de Compostela (USC), Santiago de Compostela, Spain; 3 CIBER Fisiopatología Obesidad y Nutrición (CiberOBN), Instituto Salud Carlos III, Madrid, Spain; 4 Departament of Pediatric, Universidad de Santiago de Compostela (USC), Santiago de Compostela, Spain; 5 Operative Dentistry and Endodontics, Universidad de Santiago de Compostela (USC), Santiago de Compostela, Spain; 6 Laboratorio de Endocrinología Molecular y Celular.Universidad de Santiago de Compostela (USC), Santiago de Compostela, Spain

## Abstract

Uroguanylin is a 16 amino acid peptide that constitutes a key component of the gut- brain axis with special relevance in body weight regulation. In childhood and adolescence, periods of life with notable metabolic changes; limited data exist, with measurements of pro-uroguanylin in adolescence but not in prepubertal children. This study investigates pro-uroguanylin circulating levels in children with obesity and its relationship with obesity, sex and pubertal development. We analyzed circulating prouroguanylin levels in 117 children (62) and adolescents (55), including 73 with obesity and 44 with normal weight. The pro-uroguanylin concentration is higher in lean girls during pre-puberty versus lean boys (1111 *vs* 635, p < 0.001). During puberty, pro-uroguanylin levels are higher in lean males with respect to lean females (1060 *vs* 698, p < 0.01). In girls, a negative correlation exists between pro-uroguanylin and age, Tanner stage, weight, height, BMI (body mass index), waist circumference and plasma levels of leptin and testosterone; a positive correlation was found between pro-uroguanylin and free triiodothyronine. In boys, a positive correlation was found between pro-uroguanylin and BMI and waist circumference and a negative correlation was found with high density lipoprotein-cholesterol. We conclude that a sexual dimorphism exists in circulating pro-uroguanylin levels with respect to BMI. Uroguanylin presents also an opposed circulating pattern during puberty in both sexes.

## Introduction

Obesity in children is a modern societal health problem^1^. The main implication of obesity at early ages is an increased risk of developing morbid obesity and associated pathologies during adulthood^2,3^. Obesity is a multifactorial pathology involving multiple hormones produced by peripheral tissues that act through a complex neuroendocrine system^4^. Childhood obesity is one of the most serious public health challenges of the 21st century. Overweight and children with obesity are likely to stay with obesity into adulthood and more likely to develop noncommunicable diseases like diabetes and cardiovascular diseases at a younger age. Investigation of childhood obesity therefore needs high priority.

Among the large variety of gastrointestinal derived signals, uroguanylin (UGN) has been recently proposed as a key component of the gut-brain axis involved in the regulation of energy and glucose metabolism^5^.

Uroguanylin (UGN) is a 16 amino acid peptide that is mainly secreted from the intestinal epithelium as a prohormone (pro-uroguanylin), which after cleavage yields the bioactive peptide^5–7^. UGN expression in the intestine was described in mouse^8^ and humans^9^. Different pattern of expression for UGN through the intestine was described in the different species, so in human intestine UGN is expressed in the duodenum in a small number of solitary epithelial cells of the duodenal villi^10^, however in the colon UGN is synthesized in enteroendocrine (EE) cells^11^. A recent work studied by immunohistochemistry the staining intensity for uroguanylin, guanylin and GUCY2C in children with and without obesity, showing decreased staining for uroguanylin and guanylin in girls with obesity compared to lean girls, without changes in the boys group^12^. The intestinal expression of the receptor for uroguanylin, GUCY2C was not found modified in girls or boys with obesity^12^. Studies in rodents showed that the pharmacological administration of UGN in the brain reduces feeding^13^ and weight gain in diet-induced obese mice^14^. The metabolic role of endogenous UGN is still controversial. An initial report showed that mice lacking its receptor, guanylate cyclase 2c *(Gucy*2*c)*, are hyperphagic and obese^13^, but a further study failed to find differences between mice lacking *Gucy*2*c* and wild type (WT) mice, although UGN null mice had a small increase in body weight accompanied by glucose intolerance^15^. In line with its plausible metabolic role, UGN levels were decreased in the plasma of fasted mice and recovered after refeeding or exogenous leptin infusion^16^. Consistently, circulating UGN levels were lower in leptin deficient mice and higher in hyperleptinemic diet-induced obese mice^16^. Recent findings indicate that UGN precursor (pro-UGN) is down-regulated in human obesity in adults^10^. Additionally, in a recent paper variations in pro-UGN levels were reported in adolescent girls with obesity with respect to boys without obesity^17^. All together, the mentioned data suggest that circulating levels of UGN are changed by nutritional status in a leptin dependent manner.

Nevertheless, accumulating evidence in preclinical models implicates UGN as a potential new target in obesity treatment. However, its regulation and association with key metabolic parameters in human obesity remains completely unknown. Because early pubertal children represent a stage characterized by massive metabolic changes in the context of rapid growth, we hypothesized that UGN concentrations might be deregulated in children with obesity^18–20^.

## Material and Methods

### Subjects

A total of 117 (3–17 years) Caucasian children (62) and adolescents (55), including 73 with obesity and 44 normal weight, were recruited from the Pediatrics Department at the Clinical Hospital of Santiago de Compostela. According to BMI, the sample was classified in two groups according to whether the children and adolescents had normal weight or obesity using the international BMI cut off points described by Cole *et al*.^21^. The international Cole standard is accepted by the vast majority of scientific societies for the diagnosis of overweight and obesity in children and adolescents and it is linked to the 25 and 30 cut-off points in adults. The anthropometric and analytical characteristics of these subjects are described in Table 1.

**Table 1:** Anthropometric, biochemical and hormonal characteristics of the study population.

	Lean (n = 44)	Obesity (n = 73)	*P*	*Test*
Age (yr)	9.85 ± 4.28	11.08 ± 2.83	0.233	#
Sex (girl:boy)	24:20	40:33	0.979	
Sexual development (prepuberty:puberty)	24:20	38:35	0.794	
Weight (kg)	35.97 ± 15.69	62.23 ± 20.45	<0.001	$
Height (cm)	136.82 ± 24.43	146.46 ± 23.01	0.05	$
BMI (kg/m^2^)	17.92 ± 2.98	27.25 ± 4.40	<0.001	$
Waist circumference (cm)	66.09 ± 13.05	90.97 ± 13.04	<0.001	$
Glucose (mg/dl)	79.24 ± 6.88	80.76 ± 6.72	0.251	$
Insulin (mUI/l)	7.70 ± 8.97	13.82 ± 9.42	0.006	$
IGF-1 (ng/ml)	263.38 ± 193.08	344.32 ± 196.30	0.071	$
Triglycerides (mg/dl)	63.66 ± 36.98	63.69 ± 31.32	0.997	$
Total cholesterol (mg/dl)	162.45 ± 32.59	161.44 ± 36.37	0.882	$
LDL-cholesterol (mg/dl)	96.64 ± 25.22	96.52 ± 34.96	0.986	$
HDL-cholesterol (mg/dl)	53.48 ± 15.17	47.75 ± 14.06	0.062	$
Leptin (ng/ml)	5.95 ± 7.29	18.64 ± 13.07	<0.001	$
TSH (mUI/l)	2.65 ± 1.09	2.80 ± 1.26	0.520	$
T4 (ng/dl)	1.19 ± 0.15	1.16 ± 0.14	0.257	$
T3 (pg/ml)	4.26 ± 0.49	4.20 ± 0.39	0.541	$
Estradiol (pg/ml)	31.90 ± 38.66	28.19 ± 34.84	0.654	$
Testosterone (ng/ml)	0.96 ± 1.87	0.42 ± 0.59	0.040	$
FSH (UI/l)	2.57 ± 2.16	3.02 ± 2.57	0.427	$

Values are presented as the mean ± SD. BMI, body mass index; FSH, follicle-stimulating hormone; IGF-1, insulin-like growth factor 1; TSH, thyroid-stimulating hormone; T3, triiodothyronine; T4, thyroxine. Differences in sex and sexual development distribution were analyzed by *X*^*2*^ analysis. Differences between groups were analyzed by ANOVA ($) or Mann-Whitney’s U test (#).

Enrollment into the study occurred between January 2013-October 2014. Children were excluded if they had any chronic medical conditions affecting the study results. This study was conducted according to the guidelines in the Declaration of Helsinki, and approved by the Ethics and Research Committee of the Galicia Autonomous Community under the code 2013/256. The informed consent of all children and parents and/or legal guardian (if age between 12 and 18 age) and only of the parents and/or legal guardian if age <12 were included in this study.

### Anthropometric examination and blood sampling

A clinical examination was conducted using standardized methods. The pubertal period was determined by using a gender-specific table according to Tanner’s criteria.

Clinical examinations by the pediatrics team were performed in all the patients and based in these clinical observations the corresponding Tanner stage was assigned to each patient. Pubertal stage was evaluated according to the Tanner method^22,23^, considering the stage 1 as prepubertal and stages 2 to 5 as pubertal. This method assesses breast development in girls and testicular growth in children, as well as presence of pubic hair in both genders.

Anthropometric measurements were taken in the morning with the children barefoot and in their underwear. Weight was measured with a digital electronic balance (Seca mod.813; range: 0.1–200 kg, precision: 100 g) and height with a calibrated wall-mounted stadiometer to the nearest 0.1 cm (Seca mod.213, range:20–205 cm, precision 1 mm), both from gmbh & CO. kg, Hamburg, Germany. BMI was calculated as weight (kg) divided by height squared (m^2^).

Subject blood samples were withdrawn between 0800–0900 h after 12 h of overnight fasting. The blood samples were collected into tubes containing EDTA (1 mg/ml blood). Plasma was separated by centrifugation at 4 °C and stored at −80 °C.

### Pro-UGN assay

The quantitative measurement of pro-UGN in plasma samples was performed using a commercial enzyme-linked immunosorbent assay (ELISA) kit for Human guanylate cyclase activator 2B, uroguanylin (GUCA2B, CSB-EL010048HU; CUSABIO BIOTECH CO., LTD, Wuhan, China) according to the manufacturer’s instructions. The absorbance from each sample was measured in duplicate using a spectrophotometric microplate reader at a wavelength of 450 nm (Versamax Microplate Reader; Associates of Cape Cod Incorporated, East Falmouth, MA). The assay sensitivity limit was 7.8 pg/ml. The results are presented as pg/ml.

### Western blotting

Plasma samples (dilution 1:25) were separated in 15% sodium–dodecyl sulfate-polyacrylamide gels (SDS–PAGE) and electroblotted onto polyvinylidene difluoride (PVDF) membranes (catalog 162–0177, Bio-Rad Laboratories). Equal loading of the gels and proper transfer of the proteins to the membrane were confirmed by membrane staining with Ponceau S (Sigma-Aldrich). The membranes were blocked for 1 h in 5% Bovine Serum Albumin (BSA, Sigma-Aldrich) and successively probed with anti-UGN primary antibody (dilution 1:1000) (catalog sc-67139, Santa Cruz Biotechnology INC) overnight at 4 °C and peroxide-conjugated secondary antibody for 1 h at room temperature. We used goat anti-rabbit secondary antibody (dilution 1:5000) (catalog 111–035–003, Jackson ImmunoResearch). Specific antigen-antibody binding was visualized using a chemiluminescence method according to the manufacturer’s instructions (Pierce ECL western blotting Sustrate, ThermoFisher Scientific). Image J software (NIH, Bethesda) was used to quantify the volumes of specific bands.

### Pro-UGN preadsorption

1 mg of plasma total protein was incubated with 2 µg of UGN antibody (catalog sc-67139, Santa Cruz Biotechnology INC) overnight at 4 °C, followed by addition of 20 µl of 50% protein A/G-agarose beads (catalog SC-2003, Santa Cruz Biotechnology) for 2 hours at 4 °C. After incubation, the samples were centrifuged obtaining two phases: the pellet (beads with pro-UGN immunoprecipitated) and the supernatant (sample with pro-UGN preadsorbed).

The supernatant was collected and loaded in a 15% SDS-PAGE for UGN detection by western blot and included in the ELISA kit as a negative control to determine the specificity of the assay.

### Hormonal and biochemical assays

The plasma glucose, total cholesterol and triglyceride concentrations were determined using an Advia 2400 Chemistry System (Siemens Healthcare Diagnostics, Erlangen, Germany). The low-density lipoproteins cholesterol (LDL- cholesterol) and high density lipoproteins cholesterol (HDL- cholesterol) levels were measured using a SAS-3 Cholesterol Profile kit Helena Biosciences Europe (Tyne and Wear, UK). Plasma leptin levels were quantified by an ELISA kit (DRG International, Marburg, Germany). The circulating insulin, thyroid-stimulating hormone (TSH), free triiodothyronine (T3), free thyroxine (T4), estradiol, testosterone and follicle stimulating hormone (FSH) levels were determined using chemiluminescence immunoassays (Advia Centaur XP analyzer (Siemens Healthcare Diagnostics, Erlangen, Germany)). The plasma insulin-like growth factor 1 (IGF-1) concentrations were measured by chemiluminescence immunoassays (Immulite 2000 analyzer (Siemens Healthcare Diagnostics, Erlangen, Germany)).

### Statistical Analysis

Statistical analyses were performed using the SPSS version 20.0 software statistical package (SPSS, Chicago, IL). Data are presented as the mean ± SD. Differences in the sex and sexual development distribution were analyzed by *X*^*2*^. When necessary, data were log or square root transformed to achieve a satisfactory fit to the normal distribution or variance homogeneity. ANOVA followed by SNK post-hoc test or Kruskal-Wallis test followed by Mann-Whitney U test was used for group comparison as appropriate.. The relationships between variables were analyzed by Pearson’s correlation (normally distributed data) or Spearman’s rank correlation (non-normally distributed data) coefficients (*r*). Associations between pro-UGN and the different anthropometric, biochemical and hormonal variables were evaluated using multivariate linear regression. The criterion used for selecting the best model was based on the Akaike Information Criterion (AIC) under the assumption that models with the presence of multicollinearity are rejected. The optimal model was obtained with an automatic procedure of stepwise selection using bidirectional elimination, a combination of forward selection and backward elimination, testing at each step for variables for being excluded or included. The levels of statistical significance were set at P < 0.05.

## Results

The anthropometric characteristics of the children are summarized in Table 1. Subjects with obesity exhibited significantly higher weight, BMI, waist circumference, insulin and leptin concentrations compared with age-matched and gender-matched normal weight individuals. In addition, testosterone levels were reduced in children with obesity. Normal weight and children with obesity did not differ significantly according to age, gender or sexual development (Table 1). As expected, when the study population was classified by sex, both girls and boys with obesity showed significantly higher weight, BMI and waist circumference. In addition boys with obesity had higher concentrations of insulin and leptin and lower concentrations of HDL-cholesterol compared to normal weight boys (Supplementary Table 1).

**Figure 1:**
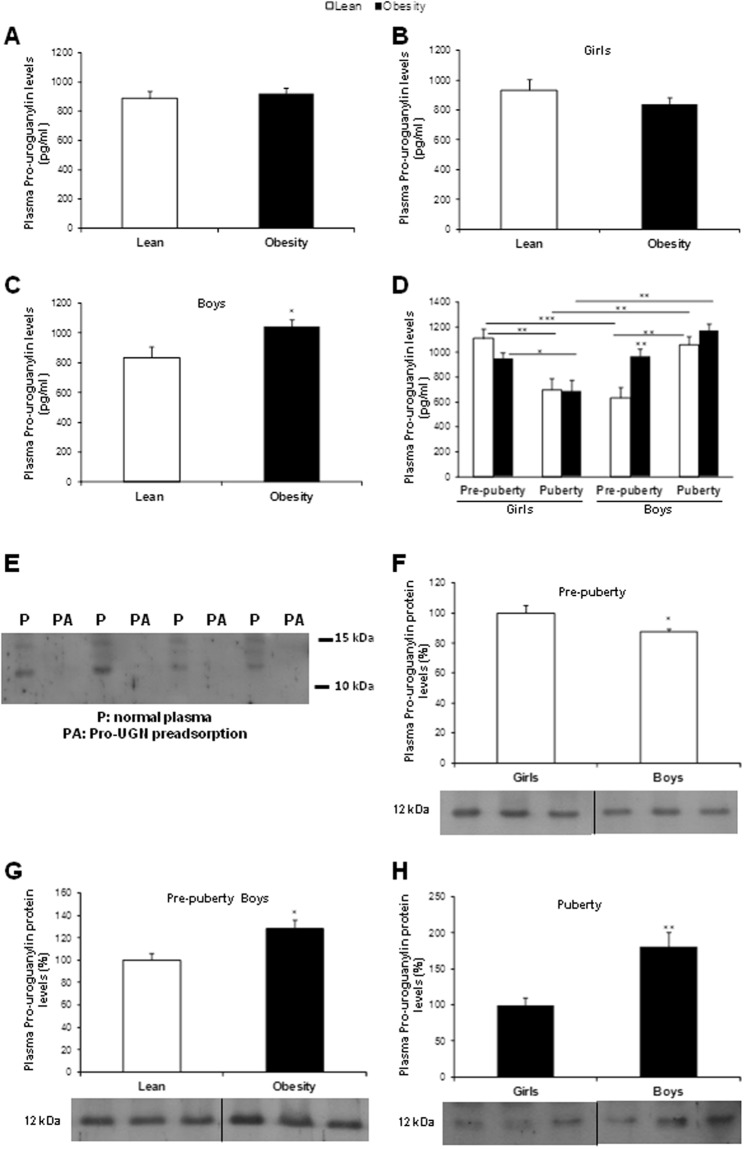
Plasma pro-UGN concentration in lean and children with obesity (**a**). Circulating levels of pro-UGN in girls (**b**) and boys (**c**). Plasma pro-UGN levels in lean and girls with obesity and boys according to sexual development (**d**). Data are expressed as the mean ± SEM. *P < 0.05; **P < 0.01; ***P < 0.001. Plasma pro-UGN protein levels measured by western blot in samples with pro-UGN preadsortion (PA) and without pro-UGN preadsorption (P) n = 4 (**e**). Plasma pro-UGN protein levels measured by western blot in pre-pubertal lean girls and boys (**f**); in pre-pubertal lean boys vs with obesity (**g**) and in puberty girls with obesity vs boys (**h**). Data are expressed as % vs control, *P < 0.05 n = 6–7.

When the total population (n = 117) was analyzed, circulating pro-UGN levels were not modified in the total population between normal weight and individuals with obesity (normal weight 890.0 ± 51.4 vs with obesity 923.9 ± 36.2 pg/ml) (Fig. 1a). To avoid the effect of a possible sexual dimorphism masking the differential regulation of pro-UGN levels by BMI, the data were independently analyzed in girls and boys. In girls with obesity, a tendency towards decreased pro-UGN levels with respect to normal weight girls was found, although it was not statistically significant (normal weight 934.0 ± 71.7 vs with obesity 838.0 ± 43.64 pg/ml) (Fig. 1b). However, boys with obesity showed a significant increase in circulating pro-UGN levels (normal weight 834.9 ± 75 vs 1046.9 ± 44.3 pg/ml) (Fig. 1c). Because sex was an important factor, we determined whether sexual development was also implicated in circulating pro-UGN levels. Therefore, we analyzed the data as a function of the sexual stage separately and found an opposing profile between girls and boys. While in the pre-pubertal stage the pro-UGN concentration was higher in lean females compared to lean males (lean females 1111.0 ± 73.5 vs lean males 635.0 ± 86.0 pg/ml), in pubertal lean males pro-UGN levels were significantly elevated with respect to lean females (lean males 1060.0 ± 67.68 vs lean females 698.0 ± 91.0 pg/ml) (Fig. 1d). When sexual maturation was achieved, the profiles were also different in boys and girls. Circulating pro-UGN levels decreased in both lean and with obesity pubertal girls in comparison to pre-pubertal girls (pre-pubertal lean girls 1111.0 ± 73.5 vs pubertal lean girls 698.0 ± 91.0 pg/ml; pre-pubertal girls with obesity 949.0 ± 45.2 vs pubertal girls with obesity 691.0 ± 87.5 pg/ml). However, pubertal boys had increased pro-UGN concentrations compared to pre-pubertal boys, even though this difference was not statistically significant in boys with obesity (pre-pubertal lean boys 635.0 ± 86.3 vs pubertal lean boys 1060.0 ± 67.68 pg/ml; pre-pubertal boys with obesity 966.0 ± 57.2 vs pubertal boys with obesity 1176.0 ± 52.3. pg/ml) (Fig. 1d).

As an additional validation for the data obtained in the immunoassay, a negative control was also measured after performing a preadsorption of the plasma samples with an anti-UGN antibody. We found that the values for circulating pro-UGN in preabsorbed samples were reduced to less than 15% (215.2 ± 76.9 pg/ml) in comparison to samples without preadsorption (1632.7 ± 221.1 pg/ml) (data not shown).

In order to corroborate the data obtained with the ELISA we also measured pro-UGN protein levels by western blot in the plasma samples of the groups that showed significant statistical differences in Fig. 1d. Firstly, plasma samples with and without preadsorption were also analyzed by western blot. We did not detect any signal in plasma where the pro-UGN was preadsorbed, but a clear signal was found for pro-UGN in plasma samples without preadsorption (Fig. 1e). Similarly to the data found by ELISA, the plasma pro-UGN protein levels measured by western blot in pre-pubertal girls were significantly elevated compared to those in pre-pubertal males (pre-pubertal girls: 100.0 ± 4.9 vs pre-pubertal boys: 87.4 ± 1.8 arbitrary units pro-UGN levels; P < 0.05) (Fig. 1f). The pre-pubertal boys group when compared lean vs boys with obesity an increased levels of pro-UGN were found in boys with obesity as showed by ELISA measures (lean boys: 100.0 ± 6.62 vs 128.5 ± 7.4 arbitrary units UGN levels; P < 0.05) (Fig. 1g). In the puberty, boys with obesity showed significantly increased protein levels of pro-UGN compared with girls with obesity (girls with obesity: 100.0 ± 9.8 vs boys with obesity: 181.2 ± 19.7 arbitrary units pro-UGN levels; P < 0.01) (Fig. 1h).

Considering the sexual dimorphism found in the present work, the correlation of circulating pro-UGN levels with the anthropometric biochemical and hormonal parameters was assessed separately in girls and boys. The correlation study in girls revealed a negative correlation between pro-UGN and age, Tanner stage, weight, height, BMI, waist circumference, leptin and testosterone. A positive correlation was found between pro-UGN and T3 (Table 2 and Supplementary Figure 1). When the correlation study was performed separately in relationship to obesity, it was found in lean girls that pro-UGN levels were negatively correlated with age, Tanner stage, weight, height, BMI, waist circumference, insulin leptin and testosterone (Supplementary Table 2). In girls with obesity, a significant negative correlation was found between pro-UGN and age and Tanner stage, and a significant positive correlation was found between pro-UGN and TSH and T3 (Supplementary Table 2). In boys, a positive correlation was found between pro-UGN and BMI and waist circumference, while a negative correlation was found between pro-UGN and HDL-cholesterol (Table 2 and Supplementary Figure 2). When the correlation study was performed separately depending on the obesity diagnostic, it was found in boys with obesity that pro-UGN correlated positively with age (Supplementary Table 2). The correlation study was also performed according to sexual development (Supplementary Table 3).

**Table 2:** Relationships of pro-uroguanylin with anthropometric, biochemical and hormonal parameters measured in the study population.

	pro-Uroguanylin			
	Girls		Boys	
	r	*P*	r	*P*
Age	**−0**.**510**	**0**.**000**	0.165	0.248
Tanner stage	**−0**.**588**	**0**.**000**	0.094	0.548
Weight	**−0**.**383**	**0**.**005**	0.286	0.054
Height	**−0**.**450**	**0**.**001**	0.066	0.663
BMI	**−0**.**278**	**0**.**048**	**0**.**334**	**0**.**023**
Waist circumference	**−0**.**396**	**0**.**004**	**0**.**342**	**0**.**020**
Glucose	−0.171	0.213	0.085	0.563
Insulin	−0.079	0.618	0.059	0.720
IGF-1	0.016	0.921	0.097	0.548
Triglycerides	0.096	0.481	−0.065	0.658
Total cholesterol	0.152	0.264	0.003	0.983
LDL-cholesterol	0.077	0.598	0.137	0.364
HDL-cholesterol	−0.094	0.519	**−0**.**291**	**0**.**045**
Leptin	**−0**.**350**	**0**.**026**	0.139	0.369
TSH	0.239	0.085	0.192	0.201
T4	−0.143	0.336	−0.241	0.119
T3	**0**.**340**	**0**.**020**	0.146	0.349
Estradiol	−0.281	0.065	0.113	0.483
Testosterone	**−0**.**34**	**0**.**023**	0.070	0.665
FSH	0.047	0.767	0.196	0.214

Statistical significance is from Pearson (normally distributed data) or Spearman (non-normally distributed data) correlation tests. Bold values indicate significant differences. BMI, body mass index; FSH, follicle-stimulating hormone; IGF-1, insulin-like growth factor 1; TSH, thyroid-stimulating hormone; T3,triiodothyronine; T4, thyroxine.

A multivariate linear regression analysis in girls and boys was performed. The optimal model in girls that best explained the pro-UGN data showed a potent positive relationship between pro-UGN and T3 and a negative association between pro-UGN and sexual development (P = 0.01) and in relationship to obesity (Table 3). For boys, the optimal multivariate linear regression model showed a positive relationship between pro-UGN and BMI and T3 (Table 4).

**Table 3:** Estimated coefficients (β) of the covariates on pro-UGN (multivariate linear regression model) with standard error (SE), t statistics and P values in girls

	β (SE)	t statistic	P value
Intercept	12.62 (4.33)	2.91	0.01
Total cholesterol	−0.31 (0.37)	−0.82	0.42
Glucose	−1.43 (0.84)	−1.70	0.10
TSH	0.08 (0.14)	0.59	0.56
T3	1.53 (0.63)	2.45	0.02
T4	−0.98 (0.50)	−1.97	0.06
Estradiol	0.07 (0.07)	0.91	0.37
Sexual development (puberty)	−0.39 (0.13)	−2.92	0.01
Diagnostic state (obesity)	−0.29 (0.13)	−2.18	0.03

TSH, thyroid-stimulating hormone; T3, triiodothyronine; T4, thyroxine.

**Table 4:** Estimated coefficients (β) of the covariates on pro-UGN (multivariate linear regression model) with standard error (SE), t statistics and P values in boys.

	β (SE)	t statistic	P value
Intercept	1.84 (2.18)	0.84	0.40
BMI	0.69 (0.26)	2.64	0.01
Total cholesterol	0.21 (0.25)	0.83	0.41
Insulin	−0.13 (0.09)	−1.51	0.14
T3	1.47 (0.63)	2.32	0.03
T4	−0.96 (0.56)	−1.72	0.09

BMI, body mass index; T3, triiodothyronine; T4, thyroxine.

## Discussion

Herein, we report the circulating levels of pro-UGN for the first time in children with and without obesity as well as its differential secretion by sex and puberty stage. Although UGN was studied for years as a natriuretic peptide^5,6^, recent preclinical studies described it as a component of the gastrointestinal-brain axis that also regulates energy and glucose homeostasis^7,13,14^. However, to the best of our knowledge, limited exists regarding circulating pro-UGN levels in individuals with obesity.

An initial comparison of the pro-UGN levels between children with and without obesity was performed in the present work, but there was no significant difference. This initial finding was unexpected taking into account that in animal models the intestinal and circulating UGN levels were tightly influenced by body composition and leptin^16^. However, when boys and girls were analyzed separately, the pro-UGN levels were similar between lean and with obesity girls, while the pro-UGN levels were higher in boys with obesity compared to lean boys. Moreover, pro-UGN was correlated negatively in girls and positively in boys with BMI. Altogether, these findings indicate sexually dimorphic pro-UGN production in children. Although no data exist about sexual dimorphism on pro-UGN circulating levels in prepubertal children, supporting the present data, a recent work reported a sexual dimorphism in the circulating levels of the UGN precursor (pro-uroguanylin) in patients with obesity with higher levels in males with obesity when compared with females with obesity^10^. However, no differences in pro-UGN levels were detected between lean males and females^10,17^. The increased pro-UGN levels found in boys with obesity are in agreement with data found in experimental animals, which showed higher UGN levels in diet-induced obese male mice^16^, but data comparing UGN in male and female rodents are not yet available. Nevertheless, the pro-satiety role of UGN in rodents (and completely unknown in humans) is still under discussion^7,13–15,24^, the scenario of higher pro-UGN levels in boys with obesity and its correlation with BMI may also suggest the existence of UGN resistance at least in children with obesity. It would be also interesting to study whether the sexual dimorphism of UGN is maintained in adults because other hormones deeply involved in energy balance, such as leptin, have shown different results in children and adults. More specifically, leptin levels are increased in adult women when compared to men^25,26^, but no differences were detected in children^27^.

Excessive adiposity during childhood may influence puberty development. In particular, exaggerated adiposity during childhood may advance puberty in girls and delay puberty in boys^28^. Therefore, for the first time we investigated the hypothesis that UGN might be influenced by puberty by comparison of pro-UGN circulating levels between pubertal and pre-pubertal population. Our cross-sectional study shows that puberty is related to changes in pro-UGN levels in boys and girls. Again, the profile in each sex is opposed; in lean pubertal girls, pro-UGN is reduced compared to pre-pubertal girls, while lean pubertal boys have increased pro-UGN levels in relation to pre-pubertal boys. Interestingly, these differences between pre-pubertal and pubertal children in both sexes are maintained in children with obesity. We also found a negative correlation in circulating pro-UGN with age and Tanner stage, supporting the hypothesis that UGN may play a role in some of the metabolic adaptations occurring at pubertal ages, raising the exciting possibility that UGN might also be physiologically involved in the maturation of the reproductive axis. Given the sexual dimorphism and the altered levels during puberty, further studies should address the role of sexual hormones on UGN levels; the potential association of UGN with endocrine-related disorders, such as polycystic ovary syndrome; and whether UGN is a primary puberty initiating signal or at least a permissive factor that allows puberty to proceed. The most recent study about this topic, is a pilot study where the authors measured pro-UGN levels in a cohort of 24 adolescent with and without obesity^17^. The authors found decreased levels of pro-UGN in girls with obesity, accordingly in the present work a negative correlation was found for girls with BMI. However the circulating levels of pro-UGN in pubertal girls with obesity were not significantly different of those in pubertal girls without obesity. In pubertal state the only difference in pro-UGN circulating levels were found with sex, being higher in boys than in girls independent of the obesity state. No differences in pro-UGN levels in boys with or without obesity were described in the previously published work^17^, accordingly in the present data pro-UGN circulating levels did not vary between pubertal boys with and without obesity.

Another novel and interesting finding is the positive correlation between T3 and pro-UGN in both girls and boys. Thyroid hormones are essential for normal growth, sexual development and reproductive function. During puberty, changes in thyroid function and an increase in thyroid volume occur as an adaptation to body and sexual development. A positive correlation between pro-UGN and thyroid hormones is shown in the present data in girls and as well as the results of multivariate linear regression analyses suggest interplay between these two hormones in both sexes.

The results obtained in the present study in childhood population open future studies in order to elucidate if the sexual dimorphism is also involved in the regulation of UGN levels in adult population. The study of a potential relationship between UGN and sexual hormones might help to understand the mechanism of UGN regulation by other physiological factors. Another interesting finding in the present paper indicates a relation between thyroid hormones and pro-UGN levels. Again, it will be of interest to investigate if this correlation is maintained in adults, as well as the biological significance of this interaction. Longitudinal studies assessing the relationship between UGN and thyroid hormones may be a more sensitive means to clarify the role of this association in puberty and its associated adaptations. The main limitation of the study is the small size of the active form UGN. The precursor of UGN is the prouroguanylina which after cleaved by a still unknown enzyme is converted to the active form. In the present manuscript, as in the the published bibliography the only form measured with the assays available in the market is the pro-Uroguanylin. Until now there are no commercial kits/antibodies in the market able to recognize only the active form UGN.

In summary, the present cross-sectional study in children demonstrates: a) the existence of a sexual dimorphism in pro-UGN circulating levels with respect to BMI, with an opposing profile between girls and boys; b) pro-UGN is oppositely released in puberty, with increased levels in boys and decreased levels in girls in comparison to pre-pubertal children; and c) pro-UGN and T3 are positively associated in both sexes.

## Electronic supplementary material

Supplemetary information

## References

[1] Ng M (2014). Global, regional, and national prevalence of overweight and obesity in children and adults during 1980-2013: a systematic analysis for the Global Burden of Disease Study 2013. Lancet.

[2] Freedman DS (2005). The relation of childhood BMI to adult adiposity: the Bogalusa Heart Study. Pediatrics.

[3] Woo Baidal, J. A. *et al*. Risk Factors for Childhood Obesity in the First 1,000 Days: A Systematic Review. *Am J Prev Med* (2016). 10.1016/j.amepre.2015.11.012 26916261

[4] Barja-Fernandez S (2015). Peripheral signals mediate the beneficial effects of gastric surgery in obesity. Gastroenterol Res Pract.

[5] Kim GW, Lin JE, Waldman SA (2013). GUCY2C: at the intersection of obesity and cancer. Trends Endocrinol Metab.

[6] Rahbi H, Narayan H, Jones DJ, Ng LL (2012). The uroguanylin system and human disease. Clin Sci (Lond).

[7] Seeley RJ, Tschop MH (2011). Uroguanylin: how the gut got another satiety hormone. J Clin Invest.

[8] Ikpa PT (2016). Guanylin and uroguanylin are produced by mouse intestinal epithelial cells of columnar and secretory lineage. Histochem Cell Biol.

[9] Brenna O (2016). Cellular localization of guanylin and uroguanylin mRNAs in human and rat duodenal and colonic mucosa. Cell Tissue Res.

[10] Rodriguez, A. *et al*. Guanylin and uroguanylin stimulate lipolysis in human visceral adipocytes. *Int J Obes (Lond)* (2016). 10.1038/ijo.2016.66 27108812

[11] Hess R (1995). GCAP-II: isolation and characterization of the circulating form of human uroguanylin. FEBS Lett.

[12] Di Guglielmo, M. D., Perdue, L., Adeyemi, A., van Golen, K. L. & Corao, D. U. Immunohistochemical Staining for Uroguanylin, a Satiety Hormone, Is Decreased in Intestinal Tissue Specimens From Female Adolescents With Obesity. *Pediatr Dev Pathol*, 1093526617722912 (2017). 10.1177/1093526617722912 PMC5647253 28847213

[13] Valentino MA (2011). A uroguanylin-GUCY2C endocrine axis regulates feeding in mice. J Clin Invest.

[14] Folgueira C (2016). Uroguanylin Action in the Brain Reduces Weight Gain in Obese Mice via Different Efferent Autonomic Pathways. Diabetes.

[15] Begg DP (2014). Effect of guanylate cyclase-C activity on energy and glucose homeostasis. Diabetes.

[16] Folgueira C (2016). Uroguanylin levels in intestine and plasma are regulated by nutritional status in a leptin-dependent manner. Eur J Nutr.

[17] Di Guglielmo, M.D., Tonb, D., He, Z., Adeyemi, A. & van Golen, K.L. A Pilot Study Measuring The Novel Satiety Hormone, Pro-Uroguanylin, In Adolescents With And Without Obesity. *J Pediatr Gastroenterol Nutr* (2017). 10.1097/MPG.0000000000001796 PMC5825243 29112082

[18] Abreu AP, Kaiser UB (2016). Pubertal development and regulation. Lancet Diabetes Endocrinol.

[19] Rogers NH (2015). Brown adipose tissue during puberty and with aging. Ann Med.

[20] Marcovecchio ML, Chiarelli F (2013). Obesity and growth during childhood and puberty. World Rev Nutr Diet.

[21] Cole TJ, Bellizzi MC, Flegal KM, Dietz WH (2000). Establishing a standard definition for child overweight and obesity worldwide: international survey. BMJ.

[22] Marshall WA, Tanner JM (1969). Variations in pattern of pubertal changes in girls. Arch Dis Child.

[23] Marshall WA, Tanner JM (1970). Variations in the pattern of pubertal changes in boys. Arch Dis Child.

[24] Folgueira C (2018). Uroguanylin: a new actor in the energy balance movie. J Mol Endocrinol.

[25] Ostlund RE, Yang JW, Klein S, Gingerich R (1996). Relation between plasma leptin concentration and body fat, gender, diet, age, and metabolic covariates. J Clin Endocrinol Metab.

[26] Brandao CM, Lombardi MT, Nishida SK, Hauache OM, Vieira JG (2003). Serum leptin concentration during puberty in healthy nonobese adolescents. Braz J Med Biol Res.

[27] Arslanian S (1998). Plasma leptin in children: relationship to puberty, gender, body composition, insulin sensitivity, and energy expenditure. Metabolism.

[28] Burt Solorzano CM, McCartney CR (2010). Obesity and the pubertal transition in girls and boys. Reproduction.

