# Supplementary material for: Circulating Pro-Uroguanylin Levels In Children And Their Relation To Obesity, Sex And Puberty
Source: Sci Rep. 2018 Sep 28;8:14541. doi: 10.1038/s41598-018-32767-7 (PMC6162323; doi:10.1038/s41598-018-32767-7)

Manuscript: CIRCULATING PRO-UROGUANYLIN LEVELS IN CHILDREN AND THEIR RELATION TO OBESITY, SEX AND PUBERTY

Authors: Cintia Folgueira, Silvia Barja-Fernández, Patricia Gonzalez-Saenz, Cecilia Castelao, Rocío Vázquez-Cobela, Veronica Pena-Leon , Manuel Ruiz-Piñon, Felipe F Casanueva, Carlos Dieguez, Rosaura Leis, Rubén Nogueiras , and Luisa M. Seoane

Table S1. Anthropometric, biochemical and hormonal characteristics of girls and boys.

	Girls						Boys					
	Lean (n= 24)		Obesity (n= 40)				Lean (n= 20)		Obesity (n= 33)			
	Pre (n=14)	Pub (n=10)	Pre (n=20)	Pub (n=20)	***P***	***Test***	Pre (n=10)	Pub (n=10)	Pre (n=18)	Pub (n=15)	***P***	***Test***
**Age (yr)**	6.3 ± 3.1^{a}	13.4 ± 2.0^{b}	9.3 ± 1.9^{c}	13.6 ± 2.1^{b}	<0.001	$	7.6 ± 2.8^{a}	13.4 ± 2.4^{b}	8.7 ± 1.8^{a}	12.9 ± 0.9^{b}	<0.001	#
**Weight, kg**	28.2 ± 15.6^{a}	49.9 ± 5.1^{b}	49.0 ± 11.1^{b}	75.7 ± 9.8^{c}	<0.001	#	20.9 ± 6.9^{a}	47.5 ± 5.3^{b}	45.3 ± 12.9^{b}	80.2 ± 21.0^{c}	<0.001	#
**Height, cm**	124.7 ± 25.4^{a}	154.2 ± 8.3^{b}	138.4 ± 10,7^{a}	158.7 ± 4.8^{b}	0.001	#	116.5 ± 18.0^{a}	155.3 ± 8.9^{b}	126.1 ± 35.4^{a}	162.4 ± 9.2^{b}	<0.001	$
**BMI, kg/m^2**	16.8 ± 2.8^{a}	21.0 ± 1.7^{b}	25.3 ± 3.7^{c}	30.0 ± 3.4^{d}	<0.001	$	14.9 ± 1.0^{a}	19.7 ± 2.1^{b}	24.1 ± 2.9^{c}	29.6 ± 4.5^{d}	<0.001	#
**WC, cm**	60.8 ± 14.8^{a}	78.0 ± 7.4^{b}	83.8 ± 8.9^{b}	98.0 ± 10.0^{c}	<0.001	$	54.1 ± 4.9^{a}	73.6 ± 5.6^{b}	81.6 ± 10.7^{b}	101.1 ± 11.9^{c}	<0.001	#
**Glucose, mg/dl**	79.8 ± 6.7	80.2 ± 6.9	79.5 ± 6.3	80.5 ± 6.7	0.978	$	77.5 ± 7.1	79.0 ± 7.6	79.0 ± 5.2	84.7 ± 8.1	0.051	$
**Insulin, mUI/l**	6.0 ± 4.0	14.2 ± 16.4	12.8 ± 10.4	16.2 ± 8.2	0.255	$	4.7 ± 3.0^{a}	6.6 ± 4.4^{a}	8.5 ± 6.5^{a}	19.0 ± 10.1^{b}	0.003	#
**IGF-1, ng/ml**	191.0 ± 178.1	357.4 ± 153.4	321.9 ± 222.5	448.8 ± 203.1	0.071	$	113.1 ± 68.6^{a}	393.8 ± 192.4^{b}	194.2 ± 43.8^{c}	409.1 ± 160.4^{b}	<0.001	#
**TG, mg/dl**	82.0 ± 46.0	61.6 ± 21.7	70.3 ± 28.7	61.0 ± 20.4	0.229	$	49.4 ± 43.2	54.5 ± 22.6	53.3 ± 31.5	72.4 ± 44.2	0.376	$
**TC, mg/dl**	161.8 ± 29.3	156.5 ± 25.8	170.0 ± 41.7	158.6 ± 24.2	0.644	$	170.9 ± 47.7	163.9 ± 28.2	158.5 ± 47.1	158.7 ± 33.5	0.876	$
**LDL-C, mg/dl**	104.1 ± 20.4	93.2 ± 16.0	104.5 ± 36.1	91.2 ± 35.5	0.548	$	99.6 ± 35.9	91.2 ± 26.5	96.9 ± 43.5	94.1 ± 25.6	0.959	$
**HDL-C, mg/dl**	45.1 ± 16.4	49.2 ± 12.2	45.3 ± 10.4	51.5 ± 19.2	0.588	$	63.4 ± 14.8^{a}	56.3 ± 12.3^{ab}	50.4 ± 13.6^{b}	43.7 ± 9.9^{b}	0.005	$
**Leptin, ng/ml**	4.2 ± 4.7^{a}	13.6 ± 7.8^{ab}	16.2 ± 7.7^{b}	28.5 ± 17.2^{c}	0.004	#	1.2 ± 0.6^{a}	6.0 ± 7.4^{ab}	11.3 ± 10.4^{bc}	17.0 ± 9.8^{c}	0.001	$
**TSH, mUI/l**	3.2 ± 1.5	2.9 ± 1.0	2.9 ± 1.3	2.3 ± 1.1	0.257	$	2.4 ± 0.8	2.3 ± 0.8	2.7 ± 1.2	3.3 ± 1.3	0.159	$
**T4, ng/dl**	1.2 ± 0.1	1.1 ± 0.1	1.2 ± 0.1	1.1 ± 0.1	0.264	$	1.3 ± 0.2^{a}	1.1 ± 0.1^{b}	1.2 ± 0.1^{ab}	1.1 ± 0.1^{b}	0.016	$
**T3, pg/ml**	4.1 ± 0.5	4.0 ± 0.4	4.2 ± 0.4	4.0 ± 0.4	0.377	$	4.6 ± 0.6	4.2 ± 0.3	4.1 ± 0.3	4.5 ± 0.3	0.061	#
**Es, pg/ml**	38.8 ± 48.4^{ab}	54.6 ± 53.6^{ab}	21.1 ± 15.6^{b}	57.4 ± 51.7^{a}	0.011	#	19.7 ± 23.8	14.2 ± 8.4	11.2 ± 8.6	16.6 ± 8.0	0.449	$
**T, ng/ml**	0.2 ± 0.2^{ab}	0.3 ± 0.1^{ab}	0.2 ± 0.1^{a}	0.4 ± 0.1^{b}	0.002	$	0.1 ± 0.0^{a}	3.3 ± 2.7^{b}	0.1 ± 0.0^{a}	1.3 ± 0.9^{b}	<0.001	#
**FSH, UI/l**	2.8 ± 1.9^{a}	3.9 ± 2.3^{a}	2.8 ± 2.3^{ab}	5.6 ± 1.8^{b}	0.002	$	0.9 ± 0.7^{a}	2.9 ± 2.1^{b}	0.9 ± 1.2^{a}	2.3 ± 1.8^{ab}	0.008	$

Values are presented as the mean ± SD. BMI, body mass index; Es, estradiol; FSH, follicle-stimulating hormone; HDL-C, HDL-cholesterol; IGF-1, insulin-like growth factor 1; LDL-C, LDL-cholesterol; T, testosterone; TC, total cholesterol; TG, triglycerides; TSH, thyroid-stimulating hormone; T3, triiodothyronine; T4, thyroxine; WC, waist circumference. Pre: prepuberty; Pub: puberty. Differences between groups were analysed by ANOVA followed by SNK post-hoc test ($) or Kruskal-Wallis test followed by Mann-Whitney U test (#). Different alphabetic superscripts represent values significantly different.

Table S2. Relationships of pro-uroguanylin with anthropometric, biochemical and hormonal parameters measured in lean and obese children.

	**Pro-uroguanylin**							
	**Girls**				**Boys**			
	**Lean**		**Obesity**		**Lean**		**Obesity**	
	r	*P*	r	*P*	r	*P*	r	*P*
**Age**	**-0.588**	**0.003**	**-0.389**	**0.023**	0.000	0.999	**0.359**	**0.047**
**Tanner stage**	**-0.622**	**0.010**	**-0.607**	**<0.001**	0.133	0.622	0.195	0.330
**Weight**	**-0.620**	**0.006**	0.125	0.480	0.234	0.365	0.213	0.267
**Height**	**-0.580**	**0.012**	-0.263	0.133	0.157	0.548	-0.011	0.956
**BMI**	**-0.755**	**<0.001**	0.049	0.783	0.440	0.077	0.056	0.774
**Waist circumference**	**-0.772**	**<0.001**	0.001	0.995	0.261	0.313	0.138	0.476
**Glucose**	-0.225	0.314	-0.132	0.463	-0.286	0.236	0.264	0.159
**Insulin**	**-0.782**	**0.013**	0.199	0.275	0.095	0.747	-0.105	0.617
**IGF-1**	-0.151	0.658	0.100	0.593	0.020	0.942	0.164	0.433
**Triglycerides**	0.009	0.967	0.136	0.443	-0.283	0.241	-0.013	0.945
**Total cholesterol**	0.419	0.052	0.018	0.920	-0.141	0.566	0.153	0.420
**LDL-cholesterol**	0.423	0.116	-0.022	0.903	0.004	0.989	0.212	0.269
**HDL-cholesterol**	0.036	0.900	-0.151	0.394	-0.388	0.124	-0.066	0.735
**Leptin**	**-0.897**	**0.002**	-0.184	0.330	0.171	0.528	-0.133	0.508
**TSH**	0.047	0.848	**0.424**	**0.012**	0.295	0.251	0.124	0.520
**T4**	-0.073	0.788	-0.195	0.294	-0.281	0.310	-0.125	0.526
**T3**	-0.193	0.491	**0.533**	**0.002**	0.122	0.665	0.278	0.151
**Estradiol**	-0.356	0.283	-0.115	0.525	0.201	0.473	0.102	0.628
**Testosterone**	**-0.767**	**0.016**	-0.158	0.381	0.129	0.660	0.071	0.731
**FSH**	0.068	0.843	0.056	0.761	0.381	0.161	0.106	0.600

Statistical significance is from Pearson (normally distributed data) or Spearman (non-normally distributed data) correlation tests. Bold values indicate significant differences. BMI, body mass index; FSH, follicle-stimulating hormone; IGF-1, insulin-like growth factor 1; TSH, thyroid-stimulating hormone; T3, triiodothyronine; T4, thyroxine.

Table S3. Relationships of pro-uroguanylin with the anthropometric, biochemical and hormonal parameters measured according to sexual development.

	**Pro-uroguanylin**							
	**Girls**				**Boys**			
	**Prepuberty**		**Puberty**		**Prepuberty**		**Puberty**	
	r	*P*	r	*P*	r	*P*	r	*P*
**Age**	-0.068	0.716	**-0.502**	**0.009**	0.135	0.493	-0.273	0.207
**Tanner stage**	-0.045	0.838	**-0.676**	**<0.001**	-0.020	0.927	-0.330	0.155
**Weight**	-0.367	0.065	-0.022	0.916	0.380	0.061	0.070	0.763
**Height**	-0.029	0.888	-0.384	0.058	-0.121	0.566	-0.246	0.282
**BMI**	**-0.448**	**0.022**	0.043	0.838	0.387	0.056	0.125	0.590
**Waist circumference**	**-0.562**	**0.003**	0.033	0.876	0.393	0.052	0.075	0.747
**Glucose**	-0.216	0.242	-0.107	0.619	-0.053	0.799	0.086	0.698
**Insulin**	-0.052	0.818	0.006	0.981	0.036	0.871	-0.116	0.668
**IGF-1**	0.000	1.000	0.277	0.223	-0.025	0.911	-0.090	0.715
**Triglycerides**	-0.115	0.540	0.244	0.239	-0.142	0.490	-0.104	0.637
**Total cholesterol**	0.273	0.137	-0.170	0.416	-0.070	0.734	0.127	0.563
**LDL-cholesterol**	0.241	0.236	-0.254	0.242	-0.005	0.983	0.411	0.058
**HDL-cholesterol**	0.105	0.610	-0.052	0.814	-0.220	0.302	-0.301	0.173
**Leptin**	-0.279	0.248	-0.079	0.748	0.269	0.204	-0.096	0.686
**TSH**	-0.025	0.901	**0.415**	**0.039**	0.183	0.371	0.258	0.272
**T4**	-0.236	0.237	-0.166	0.485	-0.228	0.263	0.079	0.763
**T3**	0.120	0.551	**0.652**	**0.002**	-0.061	0.769	0.358	0.159
**Estradiol**	**0.473**	**0.030**	-0.274	0.206	0.263	0.214	-0.305	0.234
**Testosterone**	0.000	0.999	-0.184	0.426	-0.399	0.054	-0.044	0.866
**FSH**	0.310	0.184	0.293	0.176	-0.211	0.322	0.294	0.236

Statistical significance is from Pearson (normally distributed data) or Spearman (non-normally distributed data) correlation tests. Bold values indicate significant differences. BMI, body mass index; FSH, follicle-stimulating hormone; IGF-1, insulin-like growth factor 1; TSH, thyroid-stimulating hormone; T3, triiodothyronine; T4, thyroxine.

Table S4: Pro-uroguanylin distribution among different age groups.

Age (years)	N	Pro-UGN (pg/ml)
3	4	1012.50 ± 441.34
4	5	1095.50 ± 275.19
5	4	989.50 ± 143.15
6	5	1137.0 ± 673.98
7	7	929.71 ± 196.33
8	7	883.21 ± 327.06
9	6	651.75 ± 245.66
10	19	1011.74 ± 235.63
11	10	972.00 ± 250.25
12	13	1055.65 ± 232.95
13	14	901.18 ± 369.77
14	5	558.00 ± 284.57
15	7	757.93 ± 334.64
16	1	513.00 ± -
17	1	440.00 ± -
18	1	505.00 ± -
20	1	425.00 ± -

Values are presented as mean ± SD. No significant differences between groups.

Table S5: Pro-uroguanylin distribution among different pubertal stages.

	Pubertal stages (Tanner stages)				
	**I**	**II**	**III**	**IV**	**V**
N	58	17	7	11	14
Pro-UGN (pg/ml)	963.72 ± 326.05[a]	1027.38 ± 293.04[a]	980.71 ± 324.47[a]	968.27 ± 215.24[a]	537.04 ± 266.71[b]

Values are presented as mean ± SD. Differences between groups were analyzed by an ANOVA followed by a Student-Newman-Keuls post hoc test. Different alphabetic superscripts represent values that are significantly different.

Supplementary Figure 1: Correlation between the plasma pro-uroguanylin levels and age (A), Tanner state (B), weight (C), height (D), BMI (E), waist circumference (F), leptin (G), T3 (H) and testosterone (I) in girls.

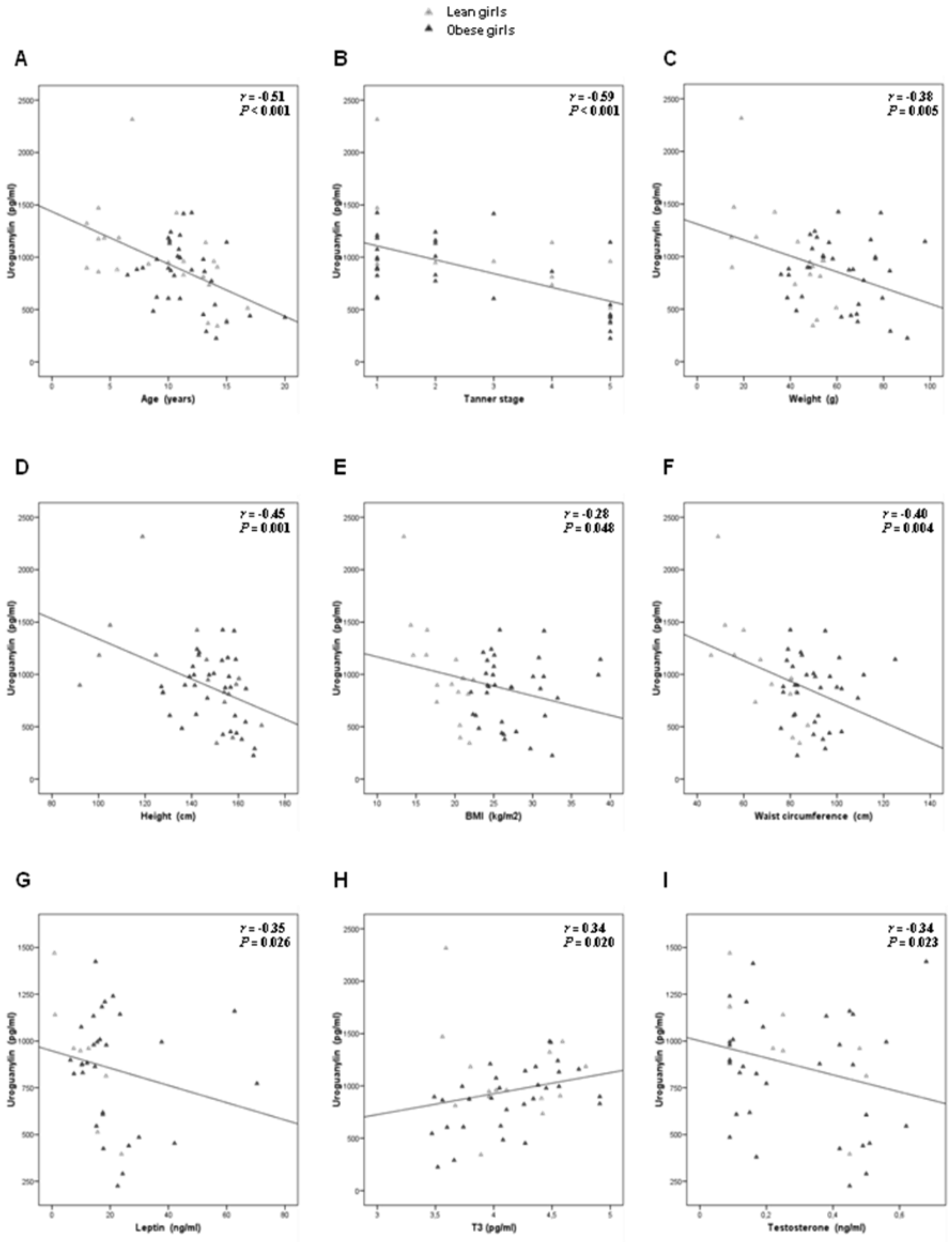

Supplementary Figure 2. Correlation between the plasma pro-uroguanylin levels and BMI (A), waist circumference (B) and HDL-cholesterol (C) in boys.

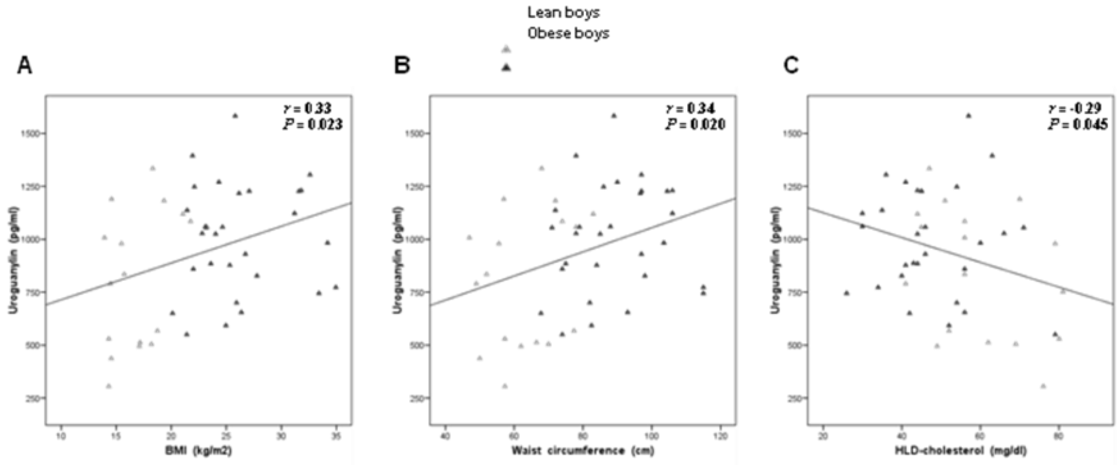

Full blots for the representative images in figures 1F, 1G and 1H that were cropped in the submitted figures:

Full blot figure 1F:

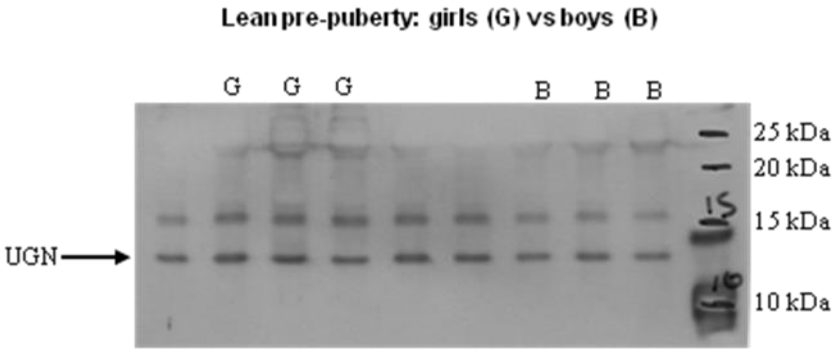

Full blot figure 1G:

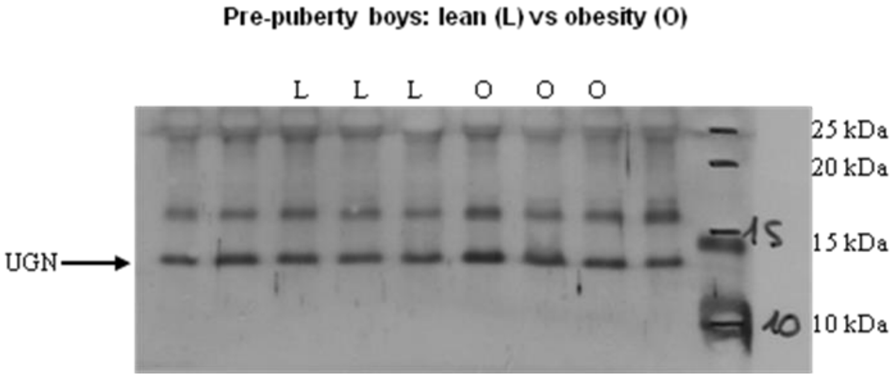

Full blot figure 1H:

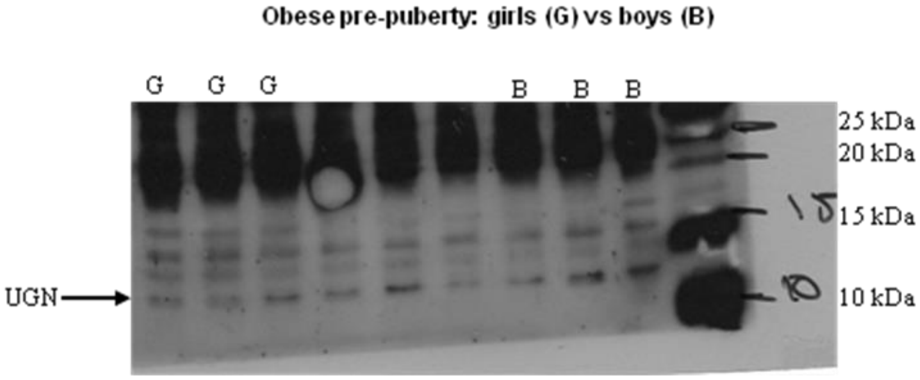

Supplement: Supplementary file 1 — Supplemetary information [file 41598_2018_32767_MOESM1_ESM.pdf]
